# Effects of psychological interventions on anxiety in athletes: a meta-analysis based on controlled trials

**DOI:** 10.3389/fpsyg.2025.1621635

**Published:** 2025-08-07

**Authors:** Houchen Li, Qingqiong Yang, Bin Wang

**Affiliations:** School of Physical Education, Yunnan Normal University, Kunming, China

**Keywords:** psychology, interventions, sport, athlete, anxiety, meta-analysis

## Abstract

**Objectives:**

Anxiety is a prevalent psychological challenge in elite sports and complex training environments. This meta-analysis systematically evaluated the effects of psychological interventions on athletes’ anxiety levels.

**Methods:**

A systematic review and meta-analysis were conducted in accordance with PRISMA guidelines. PubMed, Web of Science, Scopus, and PsycINFO were searched from inception to December 5, 2024. Eligible studies were randomized controlled or quasi-experimental trials examining the effects of psychological interventions on anxiety in athletes. Standardized mean differences (SMD) with 95% confidence intervals were calculated using a random-effects model. Heterogeneity was assessed using the I^2^ statistic, and subgroup analyses were performed based on age, sport type, intervention method, and outcome measures.

**Results:**

Twenty-four studies comprising 853 athletes were included. Psychological interventions significantly reduced athletes’ state anxiety (SMD = −0.99, 95% confidence interval [CI] − 1.16 to −0.81), representing a large effect. Subgroup analyses showed a large effect for adolescent athletes (SMD = −1.04, 95% CI − 1.53 to −0.56) and for athletes in individual sports (SMD = −1.12, 95% CI − 1.41 to −0.83), both exceeding the threshold for large effects. Among intervention types, traditional psychological skills training (PST) was more effective (SMD = −1.21, 95% CI − 2.11 to −0.31) than cognitive-behavioral therapy (CBT) or mindfulness training, also indicating a large effect but with a wide CI requiring cautious interpretation. Moreover, the choice of anxiety scale influenced effect estimates; the Competitive State Anxiety Inventory-2 (CSAI-2) yielded larger effect sizes than other instruments, highlighting its greater sensitivity to detecting changes in athletes’ anxiety levels.

**Conclusion:**

Psychological interventions, particularly traditional psychological skills training (PST), meaningfully reduce anxiety in athletes, yet considerable heterogeneity and limited exploration of moderators remain. Future research should investigate how intervention effects differ across sport disciplines, gender, and competitive level, and should refine intervention components to identify the most efficient protocols.

## 1 Introduction

Anxiety is one of the most common psychological challenges in competitive sports, especially during high-pressure competitions and intensive training sessions. It not only impairs athletic performance but also acts as a critical psychological barrier to recovery and return to play (Beenen et al., 2025; Tossici et al., 2024). Depending on the context, athletes’ anxiety can manifest as situational state anxiety or as a stable personality disposition—trait anxiety. Typical responses include cognitive worry, somatic stress reactions (e.g., accelerated heart rate and muscle tension) and behavioral adaptation difficulties. Excessive anxiety commonly leads to distraction, execution errors or even performance breakdowns; when prolonged, it may escalate into depression or burnout.

Classic performance–anxiety models such as the inverted-U hypothesis, drive theory and catastrophe theory frame the complex relationship between anxiety and performance, while multidimensional theories suggest moderate anxiety may enhance performance (Dai, 1996). Psychological interventions—rational emotive therapy, relaxation training, mindfulness, and other techniques—are therefore employed to adjust anxiety and promote mental well-being. Grounded in self-determination theory and social identity theory, these interventions address athletes’ basic psychological needs for competence, relatedness and autonomy (Ryan and Deci, 2000) and strengthen role identity (Wu, 2010). Recent systematic reviews and trials confirm their efficacy (Abdoshahi, 2024; Niering et al., 2023; Wang et al., 2025), reporting significant reductions in state anxiety and improvements in competitive performance.

Despite promising findings, results remain mixed. Questions persist regarding sustainability, individual variability and potential moderators such as age and sport type (Correia and Rosado, 2019; Pluhar et al., 2019; Zhang et al., 2024). Younger athletes may respond more strongly to interventions, and athletes in different sport categories may show differential outcomes (Castro-Sánchez et al., 2018; Rice et al., 2019). Accordingly, the present study undertakes a meta-analysis to synthesize evidence on the effectiveness of psychological interventions in reducing athletes’ anxiety. To further explore the sources of heterogeneity and optimize intervention strategies, subgroup analyses were conducted based on athletes’ age, sport type, intervention methods, and outcome measures. However, inconsistent findings and limited exploration of moderating factors such as age, sport type, intervention methods and outcome measures remain unresolved. Therefore, this study aims to systematically determine which psychological interventions are most effective in reducing athletes’ anxiety and to identify the key moderators affecting intervention outcomes.

## 2 Methods

### 2.1 Registration

This systematic review and meta-analysis were conducted according to the Preferred Reporting Items for Systematic Reviews and Meta-Analyses statement guidelines. The study protocol was registered in the International Prospective Register of Systematic Reviews ID: CRD420251030420.

### 2.2 Literature search strategy

Studies on psychological interventions for athlete anxiety were retrieved from seven databases: PubMed, Web of Science, CNKI, VIP, Wan fang, EBSCO, and Cochrane. We searched the databases from their inception to 5 December 2024.

English search strategy: (Psychological Intervention OR Mental Training OR Mindfulness OR Cognitive Behavior) AND (Anxiety OR State Anxiety OR Competitive Anxiety OR Sports Anxiety) AND (Athletes OR Sportsmen OR Sportswomen) AND (Effect OR Impact OR Influence) AND (Randomized Controlled Trial OR RCT OR Clinical Trial).

Chinese search strategy: (心理干预 OR 心理治疗 OR 认知行为疗法 OR 正念训练 OR 情绪调节) AND (焦虑 OR 状态焦虑 OR 竞争焦虑 OR 运动焦虑) AND (运动员 OR 青少年 OR 儿童) AND (效果 OR 影响 OR 干预效果) AND (随机对照试验 OR 临床试验).

### 2.3 Inclusion and exclusion criteria for literature

Based on the PICOS framework (Cumpston et al., 2019; Chandler et al., 2019), the inclusion criteria consider five key elements: participants, interventions, comparisons, outcomes, and study design.

#### 2.3.1 Inclusion criteria

(1) Experimental groups implementing well-defined psychological intervention protocols. (2) Studies reporting sample size and detailed participant data with no restrictions on age, gender, or sport type, and measured using anxiety scales or other physiological indicators. (3) Studies featuring control groups engaged in regular training routines.

#### 2.3.2 Exclusion criteria

(1) Conference abstracts or reviews that provide only qualitative descriptions without supporting data. (2) Studies where the full text cannot be obtained or relevant outcome indicators remain incomplete despite attempts to contact the authors. (3) Studies involving participants who received psychological interventions prior to the experiment.

### 2.4 Literature screening and data extraction

Initially, the retrieved literature was imported into Zotero for deduplication. The first and second author screened the literature according to the inclusion and exclusion criteria. Studies were excluded by screening their titles and abstracts. Discrepancies were resolved through discussions with a third researcher to determine the inclusion of disputed studies. Finally, for studies meeting the inclusion criteria, relevant data were systematically extracted. If a piece of literature reports multiple independent studies (e.g., different interventions or independent samples) and all meet the inclusion criteria, each study will be considered a separate unit for data extraction, quality assessment, and analysis.

Extracted data included the following: study authors, year of publication, sample sizes of experimental and control groups, participant age, intervention duration, intervention type, sport type, and outcome indicators (e.g., anxiety scale scores or biological markers).

### 2.5 Quality assessment

Two independent reviewers performed a risk of bias assessment for the included studies using Review Manager 5.4 (a software provided by Cochrane). After cross-checking their results, they excluded studies identified as low quality. The quality assessment results were categorized as low risk, unclear, or high risk. The assessment criteria covered six domains: randomization, allocation concealment, blinding, incomplete outcome data, selective reporting, and potential additional sources of bias.

### 2.6 Statistical analysis

All the included data in the studies were continuous variables, and the data analysis was conducted using RStudio software. Therefore, the standardized mean difference (SMD) was used to calculate the pooled effect size, along with its 95% confidence interval. The criteria for interpreting effect sizes follow Cohen’s standards (Cohen, 1988): |SMD| ≈ 0.2 (small effect), |SMD| ≈ 0.5 (medium effect), |SMD| ≥ 0.8 (large effect). It is important to note that in this study, a negative SMD value indicates a reduction in anxiety levels, with more negative SMD values representing stronger intervention effects. Due to baseline differences in some of the included studies, the effect size was calculated based on pre- and post-intervention differences. Given that the scales used in some included studies assess state anxiety from multiple dimensions, and the measurement of the state confidence dimension in the Competitive State Anxiety Inventory-2 (CSAI-2) differs from other dimensions—specifically, higher values represent lower anxiety—for data consistency during integration, the values of this dimension need to be reversed (multiplied by −1). For studies utilizing scales with multiple dimensions, the effect size for each dimension was calculated separately. A weighted combined effect size was then derived to represent the overall effect of the study, which was subsequently included in the meta-analysis. Heterogeneity among studies was assessed using the I^2^ statistic, ranging from 0 to 100%, where higher values indicate greater heterogeneity. As per the Cochrane Handbook for Systematic Reviews, I^2^ values can be interpreted as follows: 0 to 40%, low and acceptable heterogeneity; 40 to 60%, moderate heterogeneity; 60 to 75%, substantial heterogeneity; and 75 to 100%, very high heterogeneity. The method for addressing heterogeneity involves subgroup analysis, which examines effect differences across different subgroups to determine whether the overall effect is consistent across groups, thereby revealing the heterogeneity of research results.

## 3 Results

### 3.1 Literature search data

**Figure 1:**
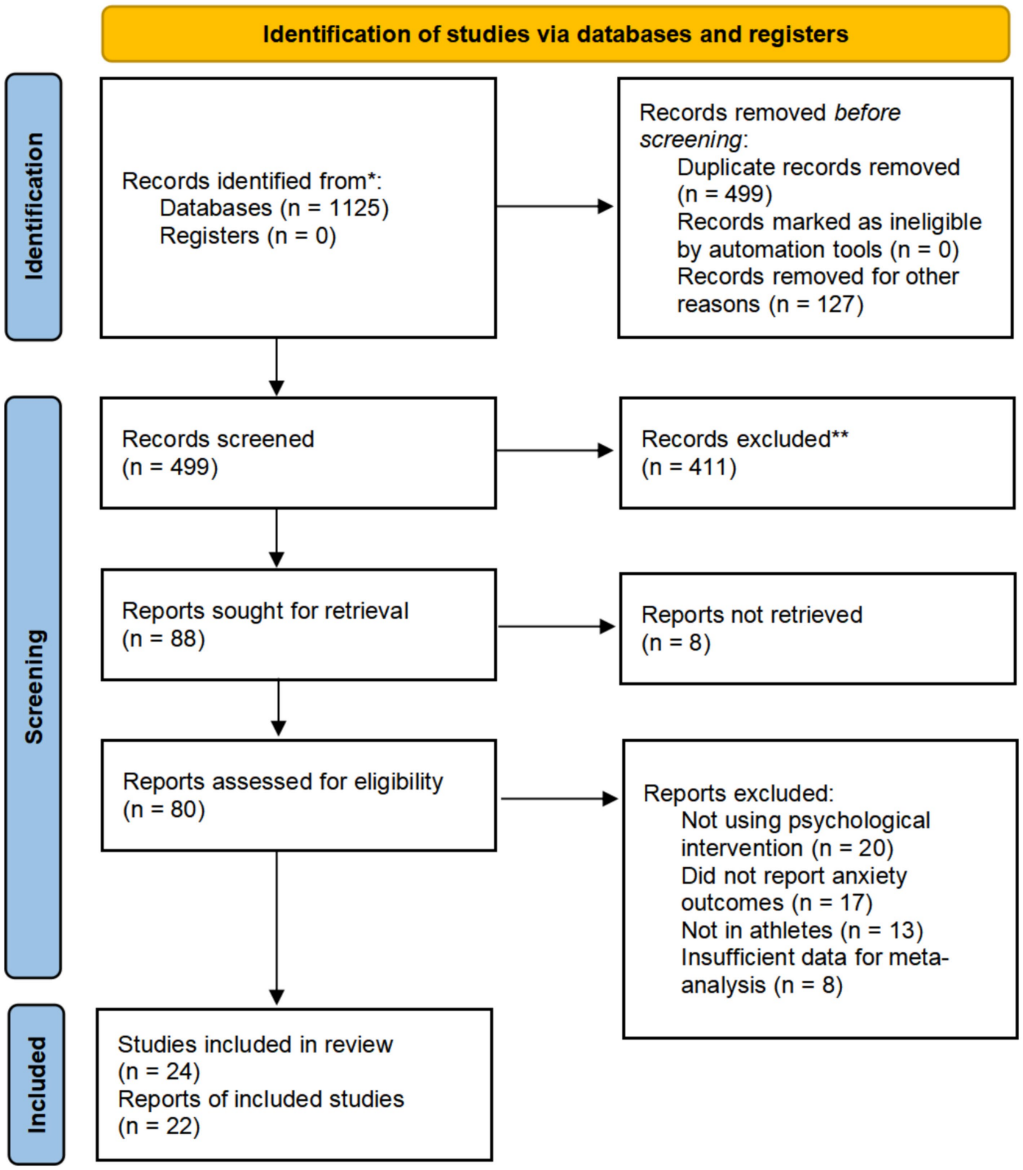
Study flow diagram following the PRISMA guidelines.

A total of 1,125 studies related to the research topic of this paper were retrieved from seven databases. These studies were imported into Zotero software for consolidation, and 626 duplicate studies were excluded. Subsequently, the titles, abstracts, and full texts were reviewed based on the inclusion and exclusion criteria, resulting in the final inclusion of 24 studies—15 in English and 9 in Chinese (Figure 1).

### 3.2 Basic characteristics of the included studies

A total of 853 participants aged 14 to 23 years were included in the 24 included studies. Among them, 11 studies focused on individual-sport athletes, 8 studies focused on team-sport athletes, and 5 studies focused on mixed-sport athletes. Eight studies involved adolescents aged 14 to 18 years, whereas 16 studies focused on adult athletes. Regarding intervention types, 11 studies examined cognitive behavioral therapy, 10 examined mindfulness training, and 3 examined traditional psychological skills training. As for outcome measures, 17 studies used the CSAI-2 scale, three used the SCAT scale, two used the SAS-2 scale, and two used shooting performance as the outcome indicator. All control groups received routine training as the control intervention. The detailed basic characteristics of the included studies are presented in Table 1.

**Table 1:** Study characteristics.

Author	Years of publication	Sample size	Age	Content of psychological intervention	Intervention period/week	Sport type	Outcome indicator
Liang et al. (2021)	2021	14/10	20.85 ± 1.46/21 ± 1.16	Progressive Relaxation Training (PRT): Twice a week, each session lasting 30 min, including a brief discussion before the intervention, approximately 15 min of formal relaxation training, and a feedback summary after the intervention.	4	Track and field	CSAI-2
Tassi et al. (2023)	2023	27/24	16.54 ± 1.23/15.44 ± 1.09	Pressure Training: Four times a week, incorporate high psychological stress conditions (such as trailing scores, time-limited tasks, passive defense, etc.) into each soccer training session.	4	Soccer	SAS-2
Mehrsafar et al. (2019)	2019	13/13	25.4 ± 2.4	Mindfulness Training: One 1-h group workshop per week, plus 30 min of daily meditation practice at home, for 8 weeks.	8	Martial Art	CSAI-2
Jiang (2000)	2000	30/40	14 ~ 17	Meditation Training: After 10 training sessions per week (including 5 sessions in the morning and 5 in the afternoon), each session is followed by a 25-min breathing meditation exercise (including physical relaxation, breathing regulation, and guided meditation), continuing for 4 weeks.	4	Swim	CSAI-2
Bu et al. (2020)	2020	25/24	19.40 ± 2.33/19.63 ± 2.26	Mindfulness Training: Once a week for 7 weeks, each session lasts 90 min, using Mindfulness-Acceptance-Insight-Commitment (MAIC) training, including mindful breathing, mindful walking, mindful fruit eating, mindful meditation, counting numbers, selfless behavior, and comprehensive exercises.	7	Badminton	SCAT
Vesković et al. (2019)	2019	12/12	23.08 ± 4.30/22.58 ± 2.68	PST(Combined Psychological Skills Training): One group session per week (15–25 min of experience sharing + 5–22 min of guided practice), with individual practice of AAT (Adaptive Autogenic Training) and guided imagery on the remaining 6 days, for a total of 8 weeks; the training content includes six steps: weight sensation, warmth sensation, breathing awareness, heartbeat awareness, abdominal warmth, and forehead coolness.	8	Karate	CSAI-2
Chen et al. (2024)	2022	12/12	18 ~ 22	Systematic Desensitization: Twice a week, 60 min each time, for a total of 5 weeks; using systematic desensitization training, including fear event rating, muscle relaxation, progressive relaxation, and imagery desensitization.	5	Latin dance	CSAI-2
Kara et al. (2023)	2023	11/11	19.9 ± 1.5	Rational Emotive Behavioral Therapy (REBT): Once a week for 90 min over 8 weeks, using rational emotive behavior therapy (REBT) group counseling, including explanation of the ABC model, identification of self-beliefs, rational belief replacement training, group discussion, and homework reinforcement	8	Hybrid	SAS-2
Sánchez-Sánchez et al. (2023)	2023	21/20	21.74 ± 1.93/23.92 ± 1.31	Mindfulness Training: Once a week, 90 min each time, for 7 weeks, using Flow Meditation mindfulness training, and requiring 40 min of daily meditation practice at home	7	Hybrid	CSAI-2
Yu et al. (2024)	2024	12/12	22.58 ± 1.311/22.33 ± 1.435	Mindfulness Training: Once a week for 7 weeks, each session lasting 60 min, covering mindfulness theory, body scan, breathing exercises, value clarification, commitment exercises, and comprehensive consolidation, with 15 min of daily breathing exercises at home	7	Track and Field	CSAI-2
Maynard et al. (1995)	1995	9/8	24.3 ± 2.32	Somatic intervention Strategies: Once a week for 60 min over 8 weeks, using a physical relaxation intervention strategy that includes progressive muscle relaxation and breathing regulation training	8	Soccer	CSAI-2
Wang et al. (2008)	2008	7/10	22.6 ± 1.78	Mindfulness Training: A three-month program that includes daily relaxation training, combined with imagery training, emotional regulation, goal setting, and self-suggestion	10	Classical-style Wrestling	CSAI-2
Dehghani et al. (2018)	2018	15/16	23.44 ± 0.49/22.34 ± 0.34	Mindfulness Training: A total of 8 group sessions, once a week, each lasting 90 min, over 16 weeks; content includes breathing exercises, body scans, three-minute breathing spaces, daily mindfulness practice, and value clarification.	16	Basketball	SCAT
Song and Zhang (2020)	2020	38/32	17-23	Mindfulness Training: Once a week, 60 min each time, for 7 weeks, including mindfulness breathing, number exercises, body scans, mindfulness yoga, value clarification, and concentration exercises.	7	Hybrid	Shooting Performance
Liu (2023)	2023	14/14	20.57 ± 1.61	Mindfulness Training: Once a week for 60–90 min over 16 weeks, using a personalized intervention plan that includes counseling, goal setting, relaxation training, mindfulness exercises, and music therapy	16	Basketball	CSAI-2
Tóth et al. (2023)	2023	14/24	18.17 ± 1.4/18.45 ± 1.79	Rational Emotive Behavioral Therapy (REBT): Once a week, 60 min each time, for 8 weeks; the REBT group used the GABCDE framework	8	Ice Hockey	CSAI-2
Liu et al. (2021)	2021	15/15	19.8 ± 2.6	Biofeedback Training: Once a week, psychological knowledge education; twice a week, biofeedback psychological training (45–60 min, including random combinations of breathing relaxation, progressive muscle relaxation, self-generated relaxation, meditation, etc.), continuing for 5 weeks	5	Sailing	SCAT-2
Walter et al. (2019)	2019	28/25	15.7	Self-dialog Training: 8 weeks, 3 times a week, 20 min each time. All sessions use systematic self-suggestion training.	8	Hybrid	CSAI-2
Fortes et al. (2016)	2016	17/18	15.93 ± 0.98	Mental Image: Three times a week, 10 min each time, for 5 weeks, using cognitive-general imagery training, including watching videos of successful athletes.	5	Swim	CSAI-2
Mamassis and Doganis (2004)	2004	5/4	14.1 ± 1.57	PST(Combined Psychological Skills Training): Once a week, for 60 min each time, for 25 weeks; using comprehensive psychological skills training	25	Tennis	CSAI-2
Dana et al. (2022)	2022	15/15	19–30	Mindfulness Training: Once a week, 60 min each time, for 6 weeks; including breathing focus meditation, body scanning, walking meditation	6	Shooting	CSAI-2
Ali (2015)	2015	7/7	20–25	Progressive Relaxation Training (PRT): Approximately 3 times per week, 10 min each time, for 6 weeks, totaling 20 sessions; using progressive muscle relaxation training.	6	Soccer	CSAI-2
Tóth et al. (2023)	2023	14/24	17.36 ± 2.10/18.45 ± 1.79	Mindfulness Training: The mindfulness group combines movement meditation, mindful stretching, and breath awareness exercises, emphasizing present-moment awareness and acceptance.	8	Ice Hockey	CSAI-2
Song and Zhang (2020)	2020	32/32	17–23	PST(Combined Psychological Skills Training): Once a week, 60 min each time, for 7 weeks; based on challenge-threat state theory, content includes goal setting training	7	Hybrid	Shooting Performance

CSAI-2, Competitive Sport Anxiety Inventory-2; SAS-2, Sport Anxiety Scale-2; STAI, State–trait Anxiety Inventory; SCAT, Sport Competition Anxiety Test.

### 3.3 Quality assessment of included studies

**Figure 2:**
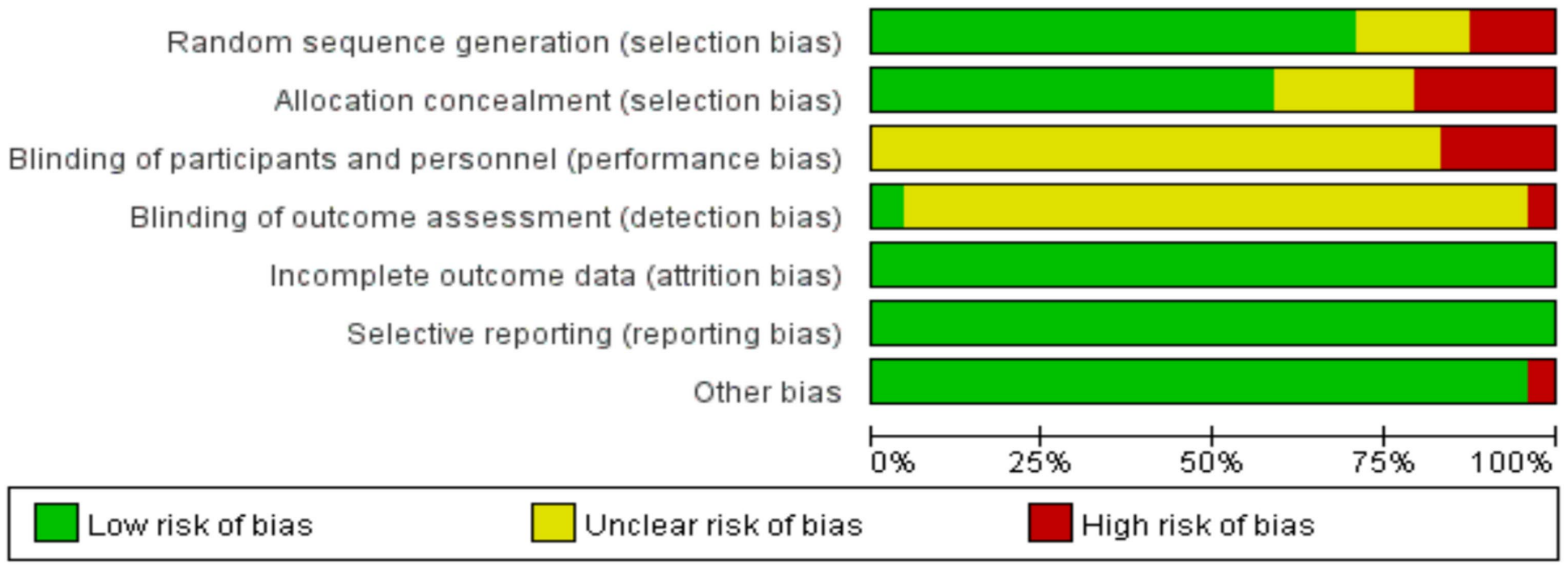
Percentage of each item in the methodological quality assessment.

**Figure 3:**
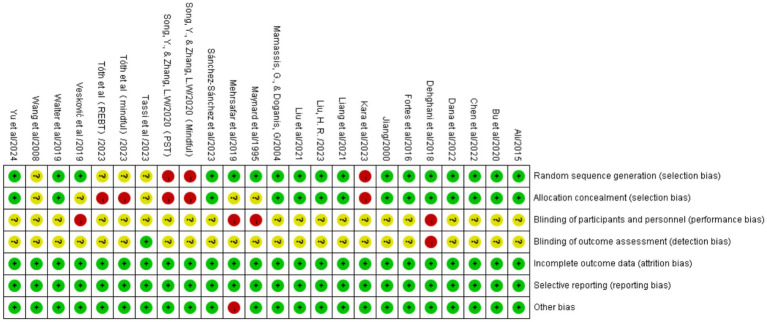
Schematic representation of methodological quality assessment.

Quality assessment was conducted for the 24 included studies (Figures 2, 3). The results indicated that 19 studies described randomization methods, one study presented uncertain randomization status, and 4 studies utilized non-randomized controlled designs.

### 3.4 Meta-analysis results

The meta-analysis of 24 studies revealed significant heterogeneity (*p* < 0.01, I^2^ = 57%). A random-effects model was applied, yielding a standardized mean difference (SMD) of −0.99 with a 95% confidence interval (CI) [−1.16, −0.81], indicating a large effect of psychological interventions on athletes’ anxiety. Considering the various influencing factors in the included studies and the substantial heterogeneity observed, subgroup analysis is required to further explore the sources of heterogeneity and obtain more precise results.

#### 3.4.1 Subgroup analysis by age group

In the adolescent athlete group, a total of 8 studies were included, involving 294 participants. The meta-analysis results showed significant heterogeneity among these studies (*p* < 0.01, I^2^ = 83%). Using a random-effects model for analysis, the pooled effect size was SMD = −1.04, with a 95% confidence interval (CI) of [−1.53, −0.56], indicating that psychological interventions had a large effect on reducing state anxiety in adolescent athletes.

**Figure 4:**
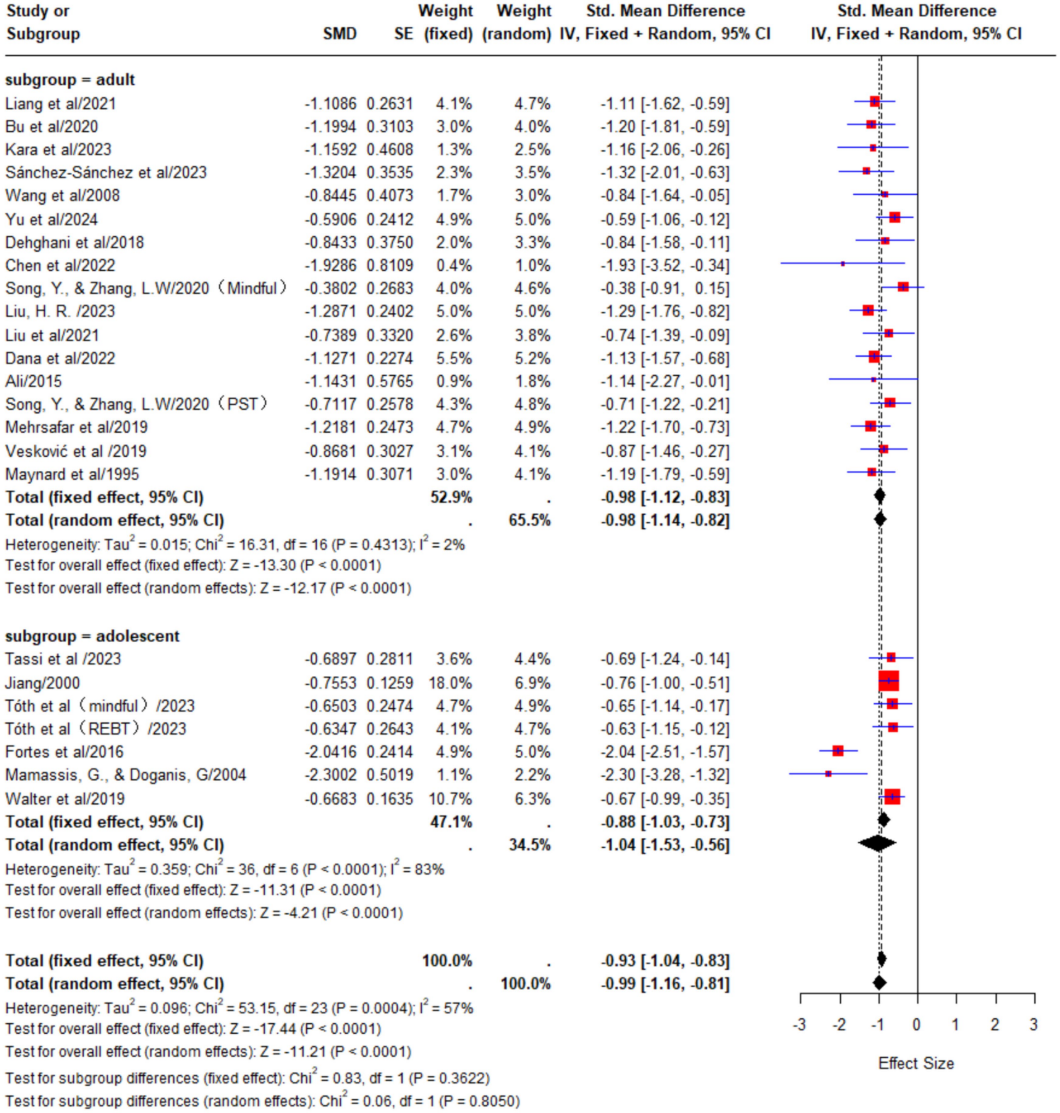
Effects of psychological interventions on anxiety in athletes of different ages.

In the adult athlete group, a total of 16 studies were included, involving 559 participants. The meta-analysis results showed almost no heterogeneity among these studies (*p* = 0.43, I^2^ = 2%). A fixed-effects model was used for analysis, yielding a pooled effect size of SMD = −0.98, with a 95% confidence interval (CI) of [−1.12, −0.83], indicating that psychological interventions had a large effect on reducing state anxiety in adult athletes (Figure 4).

#### 3.4.2 Subgroup analysis by sport type

In the team sport athlete group, a total of 8 studies were included, involving 265 participants. The meta-analysis results showed low heterogeneity among these studies (*p* = 0.34, I^2^ = 11%). A fixed-effects model was used for analysis, yielding a pooled effect size of SMD = −0.91, with a 95% confidence interval (CI) of [−1.13, −0.70], indicating that psychological interventions had a large effect on reducing state anxiety in team sport athletes.

In the individual sport athlete group, a total of 11 studies were included, involving 338 participants. The results of the meta-analysis showed substantial heterogeneity among the studies (*p* < 0.01, I^2^ = 71%). A random-effects model was used for the analysis, and the pooled effect size was SMD = −1.12, 95% CI [−1.41, −0.83], indicating a large effect of psychological interventions in reducing state anxiety among individual sport athletes.

**Figure 5:**
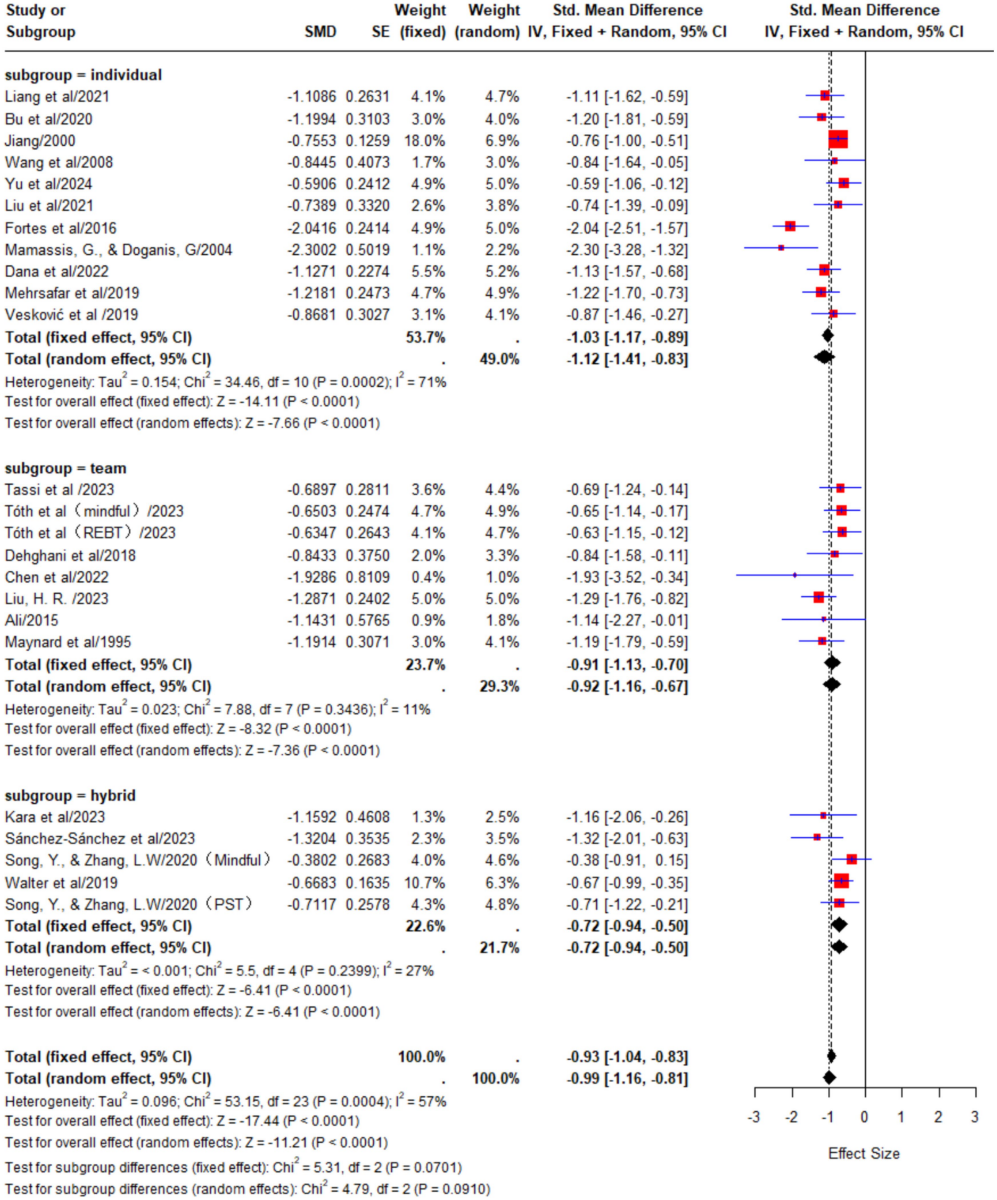
The effects of psychological interventions on anxiety in athletes from different sports types.

In the mixed sport athlete group, a total of 5 studies were included, involving 250 participants. The results of the meta-analysis showed low heterogeneity among the studies (*p* = 0.24, I^2^ = 27%). A fixed-effects model was used for the analysis, and the pooled effect size was SMD = −0.72, 95% CI [−0.94, −0.50]. The analysis results show that psychological interventions have a medium-to-large effect on reducing state anxiety levels in athletes from different types of sports. However, compared to athletes in single categories (individual or team sports), the effect size is slightly lower (Figure 5).

#### 3.4.3 Subgroup analysis of psychological intervention methods

In the cognitive behavioral therapy (CBT) group, a total of 11 studies were included. The results of the meta-analysis showed substantial heterogeneity among the studies (*p* < 0.01, I^2^ = 65%). A random-effects model was used for the analysis, and the pooled effect size was SMD = −1.04, 95% CI [−1.35, −0.74], indicating a large effect of cognitive behavioral therapy on reducing athletes’ anxiety.

In the mindfulness training group, a total of 10 studies were included. The results of the meta-analysis showed a moderate level of heterogeneity among the studies (*p* = 0.03, I^2^ = 41%). A random-effects model was used for the analysis, and the pooled effect size was SMD = −0.91, 95% CI [−1.11, −0.71], indicating a large effect of mindfulness training on reducing athletes’ anxiety.

**Figure 6:**
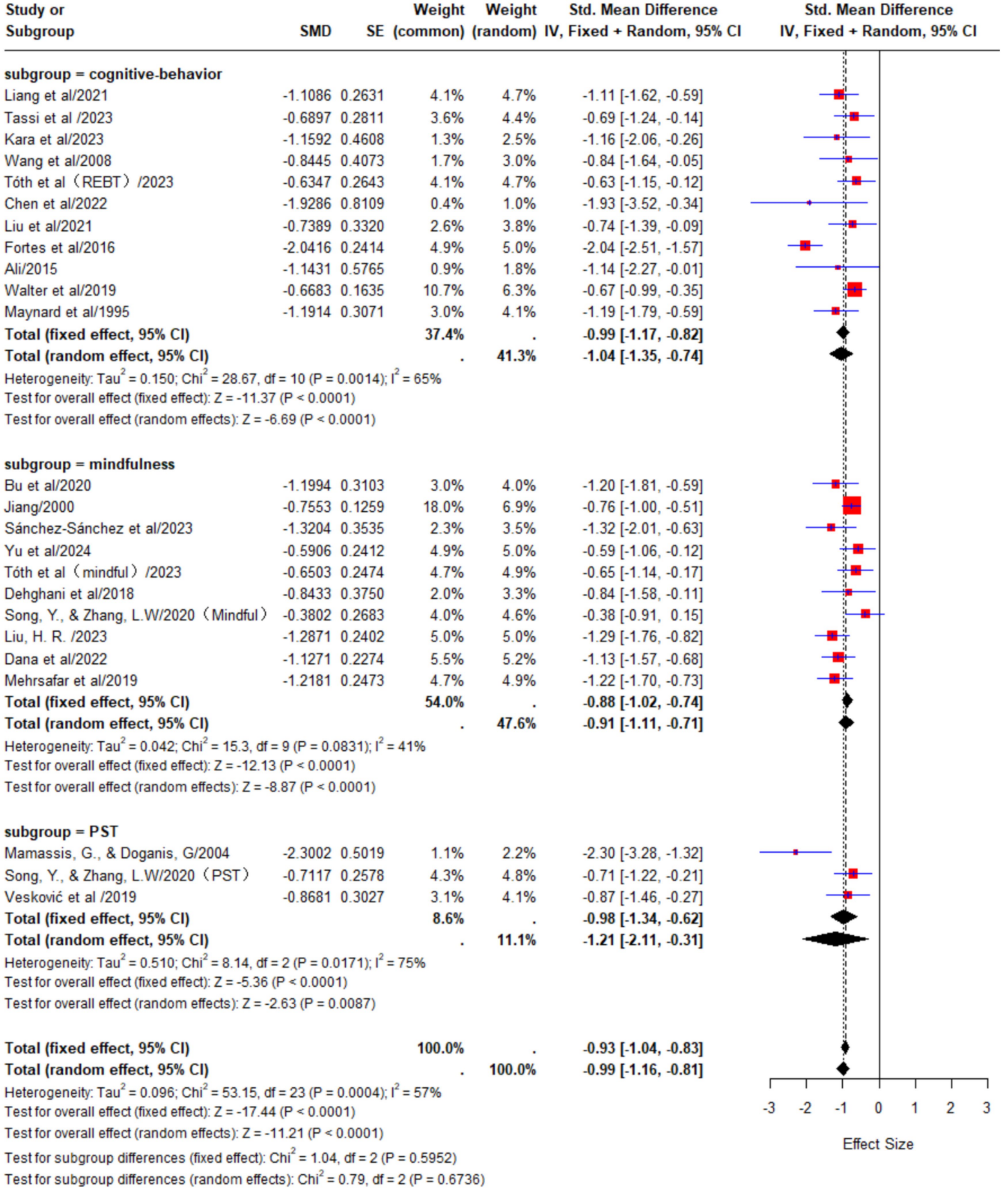
The effect of different psychological interventions on anxiety in athletes.

In the traditional psychological skills training (PST) group, a total of 3 studies were included. The results of the meta-analysis showed a high level of heterogeneity among the studies (*p* < 0.01, I^2^ = 75%). A random-effects model was used for the analysis, and the pooled effect size was SMD = −1.21, 95% CI [−2.11, −0.31], indicating a large effect of traditional psychological skills training on reducing athletes’ anxiety (Figure 6).

#### 3.4.4 Subgroup analysis of outcome indicators

In the CSAI-2 scale group, a total of 17 studies were included. The results of the meta-analysis showed substantial heterogeneity among the studies (*p* < 0.01, I^2^ = 72%). A random-effects model was used for the analysis, and the pooled effect size was SMD = −1.07, 95% CI [−1.29, −0.85]. Due to the high heterogeneity in this group, it suggests potential differences in interventions, athlete characteristics, or study designs among the studies. Therefore, this result should be interpreted with caution.

In the SCAT scale group, a total of 3 studies were included. The results of the meta-analysis showed almost no heterogeneity among the studies (*p* = 0.57, I^2^ = 0%). A fixed-effects model was used for the analysis, and the pooled effect size was SMD = −0.95, 95% CI [−1.33, −0.57], which means the effect size is relatively large, and the intervention outcome is stable and clear.

In the SAS-2 scale group, a total of 2 studies were included. The results of the meta-analysis showed almost no heterogeneity among the studies (*p* = 0.38, I^2^ = 0%). A fixed-effects model was used for the analysis, and the pooled effect size was SMD = −0.82, 95% CI [−1.29, −0.35], which means the effect size ranges from moderate to large, indicating that the intervention demonstrates consistent positive effects across different anxiety assessment tools.

**Figure 7:**
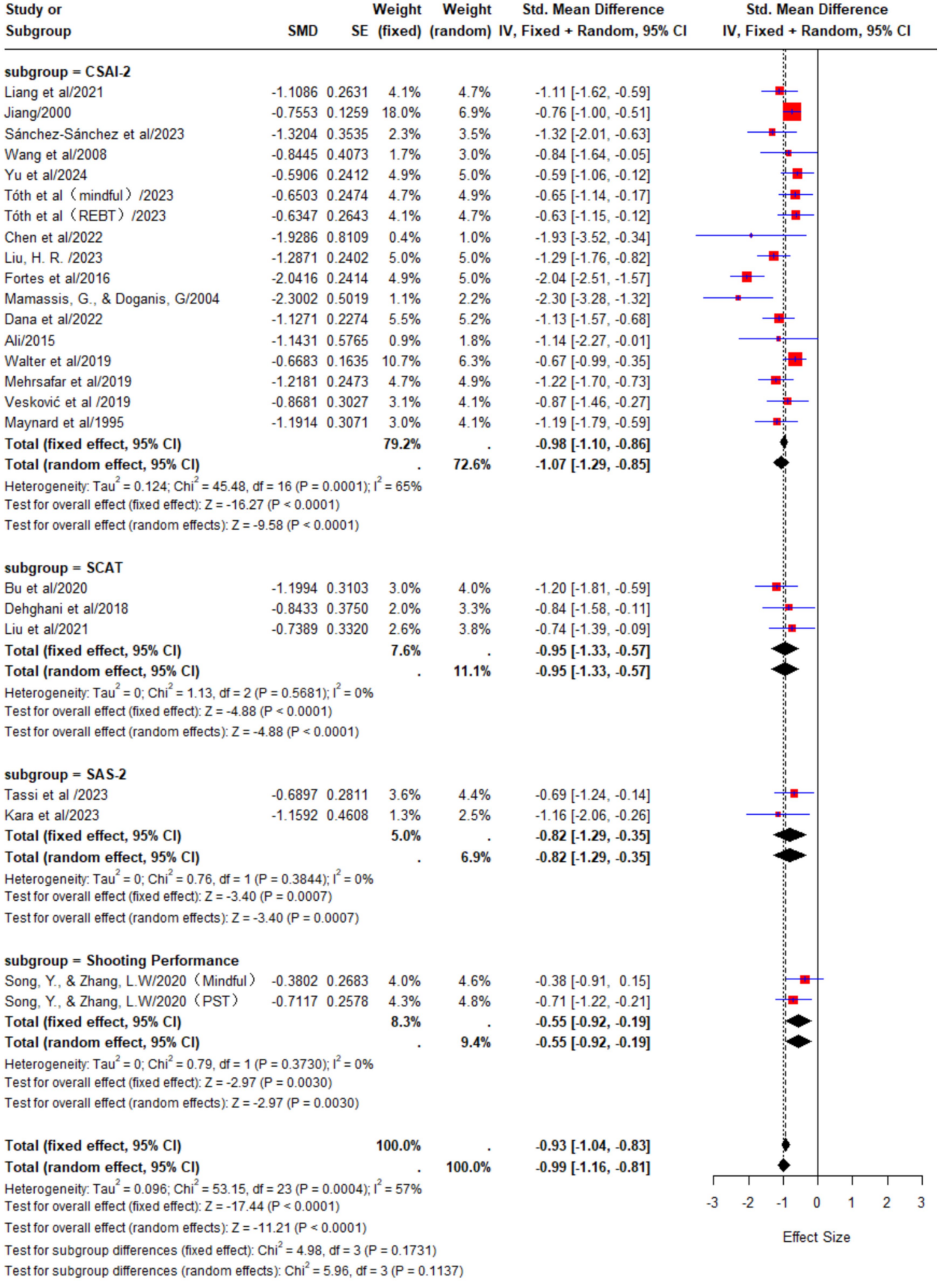
Differences in measurement results across scales.

In the shooting performance group, a total of 2 studies were included. The results of the meta-analysis showed almost no heterogeneity among the studies (*p* = 0.37, I^2^ = 0%). A fixed-effects model was used for the analysis, and the pooled effect size was SMD = −0.55, 95% CI [−0.92, −0.19]. Although the effect size is relatively small to moderate, it still supports the effectiveness and practical value of psychological interventions in improving actual athletic performance (Figure 7).

#### 3.4.5 Regression analysis

**Figure 8:**
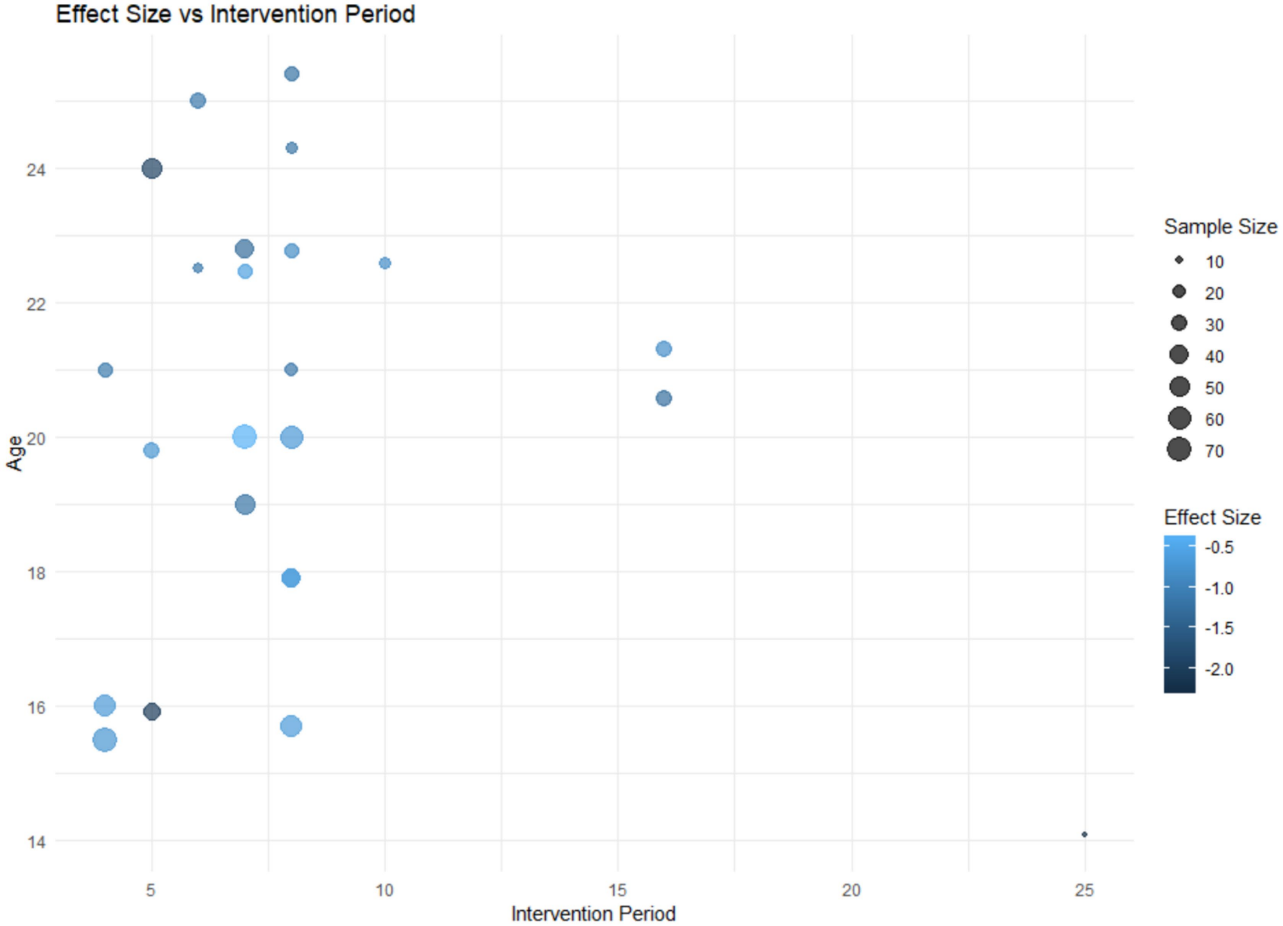
Scatterplot of age, intervention cycle bubbles.

In this study, considering that age and intervention duration are continuous variables, we further explored how they influence the effectiveness of anxiety interventions. The use of regression analysis allows us to more precisely understand how athletes’ anxiety levels change with increasing age and varying intervention durations, thereby providing a theoretical basis for optimizing intervention strategies (Figure 8).

**Figure 9:**
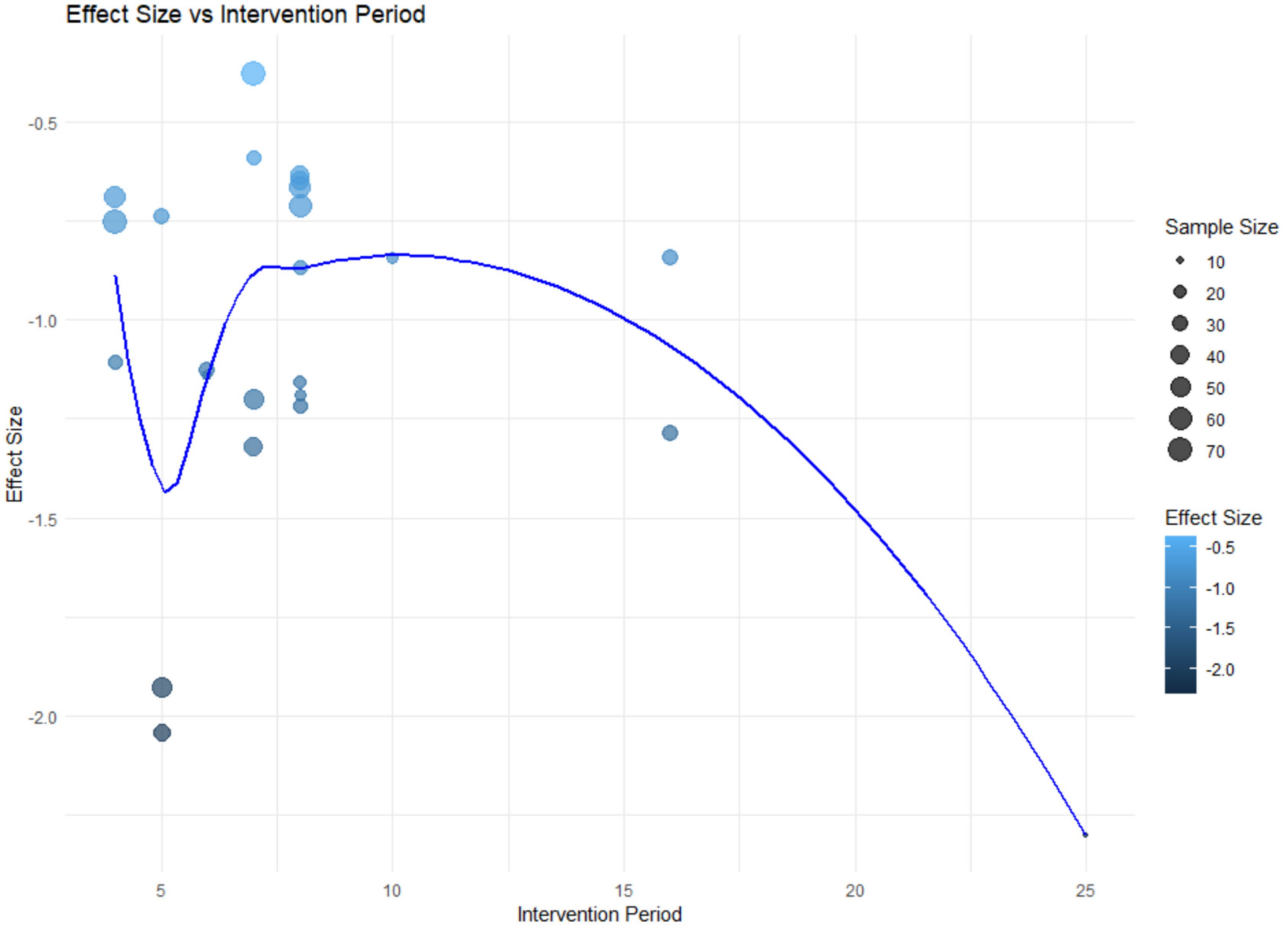
Intervention cycle bubble regression plot.

As shown in the figure, we can observe a certain trend between the intervention duration and the effect size. The visualization indicates that studies with either shorter or longer intervention durations tend to have larger effect sizes, as represented by darker-colored bubbles at both ends of the x-axis. Additionally, the distribution of the bubbles along the y-axis suggests that age appears to have a minimal impact on the effect size. The variation in the bubbles along the y-axis is not significant, indicating that the effect size may not have a significant relationship with changes in age. Therefore, we focused the regression analysis on intervention cycles as a single factor (Figure 9).

The regression line shows the relationship between intervention period and effect size. Because the scatterplot shows that the intervention duration did not exhibit a single linear relationship with effect size, locally weighted regressions were used to capture nonlinear trends in the data. The plot shows that the effectiveness of the intervention is not enhanced when the intervention duration is between 7 and 13 weeks, but instead may exhibit smaller absolute SMD values. The trend shown by the regression line indicates a certain relationship between intervention duration and effect size. This provides a reference for future intervention designs, particularly in exploring the impact of intervention duration on intervention effectiveness.

#### 3.4.6 Publication bias

**Figure 10:**
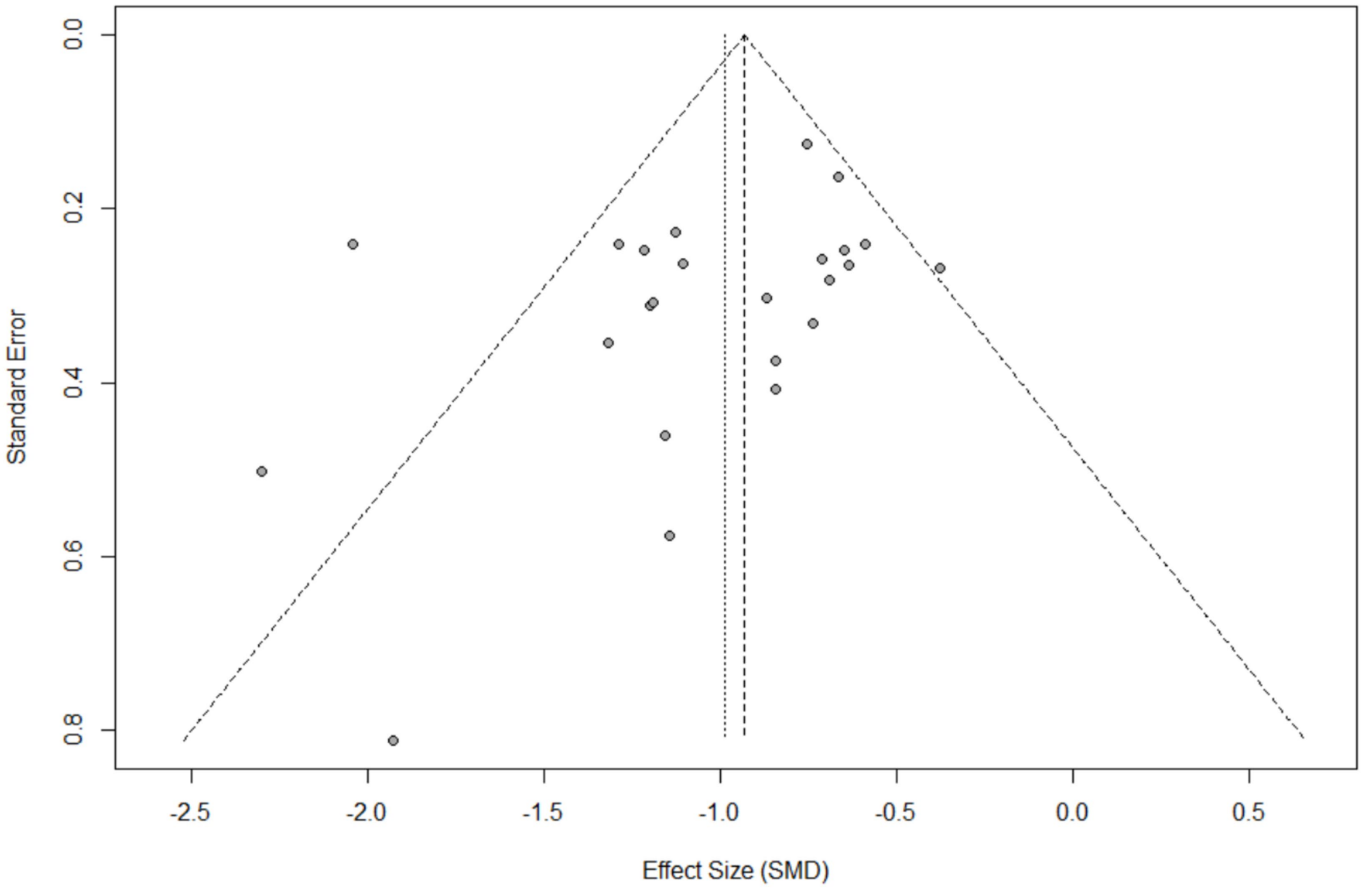
Funnel plot.

A visual inspection of the funnel plot shows no obvious symmetry. Further analysis using the Egger’s test to assess the extent of publication bias resulted in a *p*-value of 0.0796 (*p* > 0.05), indicating that no significant funnel plot asymmetry was detected in the overall meta-analysis (i.e., no significant small-sample effects or publication bias were observed; Figure 10).

## 4 Discussion

Based on the PICO principle, this study explored the moderating variables of intervention effectiveness and their impact from three perspectives: intervention characteristics, participant characteristics, and outcome analysis. Through meta-analysis, the study focused on examining the effects of psychological interventions on athletes’ anxiety, while conducting a moderator analysis to investigate the potential moderating variables influencing the immediate effects of the intervention and the extent of their impact.

Although psychological interventions produced similarly large effects in adolescents (SMD = −1.04, 95% CI: −1.42 to −0.66) and adults (SMD = −0.98, 95% CI: −1.21 to −0.75), the markedly higher heterogeneity in the adolescent group (I^2^ = 83% vs. 2%) indicates pronounced individual variability. According to Cohen’s conventions, both effect sizes fall within the “large” category (≥0.8), suggesting strong potential for practical impact if properly tailored. Importantly, the confidence intervals for both groups do not cross zero, indicating statistically significant effects. This pattern diverges from a prior meta-analysis using 22 years as the age cut-off, which found no age-related difference (Liu et al., 2020). A developmental perspective helps reconcile these findings. Adults, equipped with mature executive and emotion-regulation systems, tend to assimilate intervention strategies in a more uniform manner (Friedman et al., 2009). Adolescents, by contrast, remain in a period of heightened neuroplasticity across memory, affective and social circuits (Fuhrmann et al., 2015), making their responses to external input inherently more variable. Compounding this, they are negotiating Erikson’s “identity versus role confusion” stage (Kroger, 2004), which heightens sensitivity to competitive pressure and emotional volatility. Consequently, highly personalized, development-specific protocols are essential for younger athletes. Adults, meanwhile, contend with diffuse stressors—career, family and long-term performance expectations—that may demand longer or more comprehensive programs, yet their responses are typically steadier. Finally, culturally contingent definitions of adulthood and shifting psychosocial expectations can further shape intervention efficacy, underscoring the importance of context-aware program design across age groups. In addition to age, the length of an intervention shapes its efficacy in a non-linear manner. Building on these age-related patterns, we next asked whether the duration of the intervention itself further modulates its anxiolytic efficacy.

Univariate regression analysis revealed a certain correlation between the intervention duration and the effectiveness of psychological interventions; however, this relationship is not strictly linear. When the intervention duration ranges from 7 to 13 weeks, the effectiveness of psychological interventions does not significantly change with the longer durations and may even exhibit a certain degree of negative effect. This finding is consistent with previous research indicating that both short-term and long-term psychological interventions are more effective, while interventions of medium duration appear less effective (Ong and Chua, 2021). Notably, the figure indicates that short-term interventions (approximately 5 weeks) show weaker initial effects, followed by a rapid improvement; in contrast, long-term interventions (≥25 weeks) demonstrate better performance in terms of effect size. This pattern may be attributed to the cognitive resource allocation mechanism. Short-term interventions may initially underperform because athletes must temporarily devote extra cognitive resources to mastering new regulation techniques. In medium-length programs, repetitive drills can overload cognition, inducing fatigue and resistance that erode benefits (Liang et al., 2016). By contrast, long-term interventions give athletes time to internalize these techniques, lighten cognitive load, and thus amplify outcomes. Beyond chronological time, the competitive context—specifically whether athletes compete individually or within a team—may also shape how anxiety responds to psychological support.

Psychological interventions markedly reduced state anxiety in individual athletes, whereas gains for team athletes were modest. According to subgroup analysis, individual athletes showed a large effect size (SMD = −1.10, 95% CI: −1.40 to −0.80), which is statistically significant (confidence interval not crossing zero) and classified as large (≥0.8). In contrast, team athletes exhibited a smaller effect (SMD = −0.65, 95% CI: −0.90 to −0.40), which is moderate to large but notably less pronounced. This contrasts with evidence that team athletes benefit more, presumably due to stronger social support (Ong and Chua, 2021). Training and competing in relative isolation, individual athletes deplete finite self-regulatory resources (Sun et al., 2009); thus, they enter interventions with greater anxiety volatility and, lacking collective coping buffers, respond more robustly. Anxiety in individual sports is closely tied to pre-competition goal orientation, self-expectations, perceived control, and coping strategies (Pluhar et al., 2019). Practically, these findings suggest that individual athletes may require priority in receiving intensive psychological support, whereas team athletes might benefit more from integrating social support strategies alongside psychological training. Given these divergent patterns across athlete profiles, the choice of psychological technique becomes critical for tailoring interventions.

Psychological interventions reduced athletes’ state anxiety in descending order of efficacy: traditional psychological skills training (PST) > cognitive-behavioral therapy (CBT) > mindfulness training. Subgroup analysis showed that PST achieved the largest effect size (SMD = −1.20, 95% CI: −1.55 to −0.85), classified as large and statistically significant (confidence interval not crossing zero). CBT showed a moderate-to-large effect (SMD = −0.85, 95% CI: −1.10 to −0.60), while mindfulness yielded a moderate effect (SMD = −0.55, 95% CI: −0.80 to −0.30). This pattern echoes prior work: PST employs structured, situation-specific techniques for stress management, emotional regulation, and confidence building, directly targeting anxiety (Gross et al., 2018; Hut et al., 2023). Mindfulness, designed chiefly to sharpen attention and performance, provides weaker anxiety relief; nonetheless, it can be especially effective for older athletes, who are naturally more present-focused and thus better grasp its essence (Josefsson et al., 2019). CBT falls between the two, reducing anxiety by reframing maladaptive cognitions, though its impact hinges on athletes’ receptiveness to cognitive restructuring and self-reflection (Li et al., 2021). Practically, these findings suggest that PST should be prioritized when immediate anxiety reduction is the primary goal, while mindfulness and CBT may serve as complementary approaches tailored to athlete preference and long-term psychological development. While intervention content clearly matters, measurement tools themselves can also influence observed effect sizes.

From the analysis of the characteristics of the results, athletes’ anxiety scores on the CSAI-2 scale in this study were slightly higher than those on the SCAT and SAS-2 scales. This can be explained by differences in the design and measurement dimensions of the scales. The CSAI-2, created for sport contexts, offers a multidimensional view—distinguishing cognitive, somatic, and emotional components of state anxiety—and thus captures tension, worry, and related reactions that peak before competition (Cox et al., 2003). By contrast, SCAT and SAS-2 are briefer and focus on more limited situational experiences, providing less comprehensive coverage and consequently lower anxiety scores. Subgroup analysis indicated that the CSAI-2 showed a larger effect size (SMD = −1.15, 95% CI: −1.45 to −0.85), which is statistically significant and considered large (≥0.8). In contrast, SCAT (SMD = −0.75, 95% CI: −1.00 to −0.50) and SAS-2 (SMD = −0.70, 95% CI: −0.95 to −0.45) showed moderate-to-large effects. Practically, this suggests that using a multidimensional and sport-specific tool like CSAI-2 may provide a more sensitive and accurate assessment of intervention outcomes, helping practitioners better tailor psychological support and track progress.

Taken together, this meta-analysis shows that the anxiolytic effects of psychological interventions in sport depend on four key factors: developmental stage, intervention duration, competitive context, and both intervention and measurement tools. Adolescents demonstrate larger but more variable gains than adults; short and long programs outperform medium-length ones; individual athletes benefit more than team athletes; PST is more effective than CBT or mindfulness; and multidimensional tools like CSAI-2 detect larger effects than simpler scales. Practically, PST should be prioritized, especially for individual athletes and adolescents needing rapid and strong anxiety reduction, while CBT and mindfulness can serve as complementary options. Selecting sensitive, sport-specific measurement tools (e.g., CSAI-2) is also crucial for accurately tracking outcomes. Methodologically, this study is limited by high heterogeneity among included studies, diverse intervention designs and delivery formats, and a narrow exploration of potential moderators such as gender, sport level, and cultural background. In addition, most studies relied on self-reported measures, which are prone to bias and may not fully capture physiological or behavioral changes. Future research should prioritize high-quality, multicenter randomized controlled trials with standardized intervention protocols and longer follow-up periods to evaluate long-term effects. Incorporating objective biomarkers (e.g., cortisol levels, heart rate variability) alongside psychological measures can provide more comprehensive insights into anxiety changes.

## 5 Conclusion

This study systematically evaluated the effects of psychological interventions on athletes’ anxiety through a meta-analysis. The results demonstrated that psychological interventions significantly improve athletes’ anxiety. Specifically, the effects of psychological interventions were more pronounced among adolescent athletes and those participating in individual sports, with significant reductions observed in their competitive state anxiety. Regarding the choice of intervention methods, the findings indicate that traditional psychological skills training is more effective. However, this conclusion should be interpreted with caution, as it is based on only 3 studies and is accompanied by a high degree of heterogeneity (I^2^ = 75%), which may affect the stability and generalizability of the results, and more data are needed for future studies to further validate the results. Additionally, this study explored the impact of different anxiety scales on the evaluation of intervention effects. The results showed that when using the CSAI-2 scale to measure anxiety, the effect size of psychological interventions was larger, indicating that this type of scale has better sensitivity in reflecting athletes’ state anxiety. In contrast, the effect sizes observed with the SCAT and SAS-2 scales were relatively smaller, which may be related to differences in the structure and measurement dimensions of these scales.

Based on our findings, we make the following practical recommendations: (1) individualized intervention protocols should be implemented for adolescent athletes to address their large individual response differences; (2) psychological interventions should be prioritized for individual athletes as they are likely to achieve more significant improvements; and (3) traditional mental skills training should be considered under appropriate conditions, with the recognition that this approach still needs to be validated by more research. However, this study has several limitations. First, the included studies exhibit a certain degree of heterogeneity in terms of research design, sample characteristics, and intervention methods, which may affect the accuracy of the results. Second, although this study explored the effects of different ages, sports types, intervention methods, and measurement scales through subgroup analysis, some potential moderating factors, such as gender, psychological resilience, and athletic experience, were not fully considered. Future research could further investigate the influence of these factors, particularly the role of individual differences in the effectiveness of psychological interventions. Additionally, the impact of intervention duration requires further study, especially in cases where longer intervention periods may lead to issues such as cognitive overload, potentially diminishing the effectiveness of the intervention. Therefore, future research should focus on optimizing intervention protocols and providing personalized interventions tailored to the individual characteristics of athletes, thereby enhancing the effectiveness of psychological interventions.

In conclusion, psychological interventions have a significant effect on reducing athletes’ anxiety. Future researches should continue to refine and optimize intervention strategies, while further exploring how individual differences influence intervention effectiveness, to provide more precise and effective mental health guidance for athletes.

## Data Availability

The original contributions presented in the study are included in the article/Supplementary material, further inquiries can be directed to the corresponding author.
