# Supplementary material for: Effects of psychological interventions on anxiety in athletes: a meta-analysis based on controlled trials
Source: Front Psychol. 2025 Aug 7;16:1621635. doi: 10.3389/fpsyg.2025.1621635 (PMC12368976; doi:10.3389/fpsyg.2025.1621635)
Supplement: Supplementary file 1 [file Data_Sheet_1.PDF]

Effects of psychological interventions on anxiety in athletes: A meta-analysis based on controlled trials

BACKGROUND: Anxiety is one of the most common psychological challenges in competitive sport, especially during high-pressure competitions and training. Although many studies have examined the ability of psychological interventions to alleviate anxiety and enhance performance, controversy and uncertainty remain regarding the persistence of their effects, individual differences (e.g., possible effects of age, type of sport)

All are athletes and included between the ages of 14 and 23, with no restrictions on sports

A search of PubMed, Web of Science, CNKI, VIP, Wan fang, EBSCO, and Cochrane included 24 studies on the effects of psychological interventions on anxiety in athletes

- ✓ Experimental groups implementing well-defined psychological intervention protocols
- ✓ Studies featuring control groups engaged in regular training routines
- ✗ Studies where the full text cannot be obtained
- ✗ Studies involving participants who received psychological interventions prior to the experiment

	psychological interventions	conventional control
Anxiety	SMD=-0.99[-1.16,-0.81]	P<0.01

Subgroup analyses:sports,age,phychological intervention and anxiety measurement tools;Meanwhile regressions were also conducted on intervention duration to observe the relationship between intervention effects and duration

CONCLUSION: Psychological state significantly reduces anxiety levels in athletes. Adolescent and individual program athletes' showed greater gains in psychological interventions, with traditional skills training (PST) showing the greatest effect. In addition, greater intervention effects were observed when anxiety was measured by the CSAI-2 scale. The regression line suggests that the effect of the intervention does not increase when the duration of the intervention is between 7 and 13 weeks, but may instead show smaller values.

Li et al.
